# A Status Report on “Gold Standard” Machine-Learned Potentials for Water

**DOI:** 10.1021/acs.jpclett.3c01791

**Published:** 2023-09-01

**Authors:** Qi Yu, Chen Qu, Paul L. Houston, Apurba Nandi, Priyanka Pandey, Riccardo Conte, Joel M. Bowman

**Affiliations:** † Department of Chemistry and Cherry L. Emerson Center for Scientific Computation, Emory University, Atlanta, Georgia 30322, United States; ‡ Independent Researcher, Toronto, Ontario M9B 0E3, Canada; ¶ Department of Chemistry and Chemical Biology, Cornell University, Ithaca, New York 14853, United States; ∇ Department of Chemistry and Biochemistry, Georgia Institute of Technology, Atlanta, Georgia 30332, United States; § Department of Chemistry and Cherry L. Emerson Center for Scientific Computation, Emory University, Atlanta, Georgia 30322, United States; ∥ Department of Physics and Materials Science, University of Luxembourg, L-1511, Luxembourg City, Luxembourg; ⊥ Dipartimento di Chimica, Università degli Studi di Milano, via Golgi 19, 20133 Milano, Italy

## Abstract

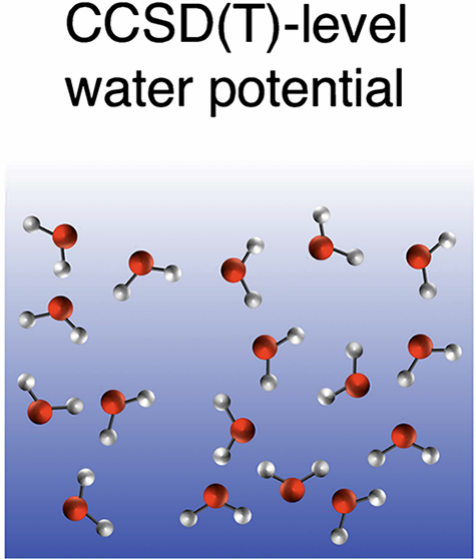

Owing to the central importance of water to life as well as its unusual properties, potentials for water have been the subject of extensive research over the past 50 years. Recently, five potentials based on different machine learning approaches have been reported that are at or near the “gold standard” CCSD(T) level of theory. The development of such high-level potentials enables efficient and accurate simulations of water systems using classical and quantum dynamical approaches. This Perspective serves as a status report of these potentials, focusing on their methodology and applications to water systems across different phases. Their performances on the energies of gas phase water clusters, as well as condensed phase structural and dynamical properties, are discussed.

Arguably, the most important component of nuclear dynamics simulations (broadly defined) of molecules, clusters, and condensed phase molecular systems is the potential energy surface (PES), also termed the force field (FF). We recall that a “first-principles” PES makes explicit use of Born–Oppenheimer separation of electronic and nuclear motion. The former describes the PES and the latter the nuclear dynamics. Since the PES is derived from electronic motion, it represents the most quantum mechanical aspect of the overall simulation. While it would be ideal to describe nuclear dynamics quantum mechanically, this is prohibitive for applications involving condensed phases. Fortunately, classical molecular dynamics (MD) and path-integral motivated MD approaches provide an accurate description of the dynamics currently of interest, such as the diffusion constant. For thermodynamic and dynamic properties of hydrogenic molecules and materials, rigorous path-integral methods are widely employed to capture substantial nuclear quantum effects. For non-hydrogenic materials, classical MD is generally adequate. In any case, the ultimate accuracy of dynamics and statistical mechanical simulations depends critically on the accuracy of the PES. We conclude this introductory paragraph by noting that the current “gold standard” quantum chemistry approach for the PES is the coupled-cluster method, with CCSD(T) being the current workhorse version. So this method is used synonymously with “gold standard”. However, this level of electronic structure theory is also very computer-intensive with scaling of O(*N*^7^), where *N* is the size of the electronic space. So clearly this method cannot currently be extensively applied to molecules or clusters of more than roughly ten atoms.

These general remarks are applicable to PESs describing water, from the smallest water dimer cluster to systems encompassing hundreds of water monomers. The motivation behind simulating the unique properties of water is widely recognized and does not require reiterating. Thus, it is not surprising that there are over 50 PESs/FFs available that describe noncovalent water interactions. One general aim of these PESs is to treat an arbitrary number of monomers and perforce the use of simple functional forms. The historically important ones, which are still the mostly widely used, are based on simple electrostatic models with two-body noncovalent potentials with parameters determined empirically. These potentials are limited in the underlying chemical physics and, of course, are not “first-principles”. Such approaches have been intensively investigated in the past decade using density functional theory (DFT) and initially using direct dynamics, which by-passes the need for a functional representation of the PES.^1−4^ DFT is the only electronic structure method that is feasible for these calculations; however, it still involves a large cost in computational effort. Some interesting efforts to improve DFT using the many-body CCSD(T)-based MB-pol have been reported.^5^ DFT-based machine learning potentials have also recently appeared.^6−10^

Ideally, one would like to have the best of both worlds, namely, an analytical PES for water, based on *ab initio* electronic energies (and possibly forces). The “holy grail” of this effort would be for the energies to be at the gold-standard CCSD(T) level. There has been great progress toward achieving this goal very recently using machine-learning approaches, and that is the focus of this Perspective. To be clear about this progress, and to preface the outline of the paper, we state at the outset that the progress is based on two different approaches, neither of which is based on direct fitting of CCSD(T) energies (and forces) for tens or hundreds of water monomers, as this would be totally infeasible to do. One approach is the many-body expansion, and the other, more recent one is based on transfer learning to bring a DFT-based potential close to the CCSD(T) level. We begin with the many-body expansion approach.

## Many-Body Approaches

### q-AQUA

To begin, we note that the strict many-body expansion (MBE) for the total energy of *N* water monomers is given by
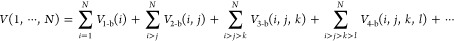
1where *V*_1-b_ denotes the 1-body potential, i.e., the potential for the isolated monomer, and *V*_2-b_, *V*_3-b_, *V*_4-b_, etc. are the *intrinsic* 2-, 3-, 4-body, etc. interactions. This representation, truncated at the 4-b term, was used extensively in seminal studies of the binding energies at the MP2 and MP4 level of theory for moderate sized clusters, from the dimer to the hexamer in 1994.^11^ From this early work, it was concluded that the expansion converged at the 3-body level and that 4-body terms were “negligible”. This conclusion was essentially confirmed in recent work by Heindel and Xantheas,^12,13^ who extended the analysis to clusters as large as the 21-mer. With basis set superposition errors eliminated, it was concluded that the 4-body interactions account for at most one percent of the total binding energy. The slight change in the assessment of the four-body interaction from “negligible” to “very small” is probably a result of the increasing number of 4-b terms for the larger clusters studied in the more recent study. This important aspect of the MBE was discussed in detail in a 2010 Frontiers Article.^14^ It was noted that, although the number of four-body interactions for *N* monomers goes as *O*(*N*^4^), this is quantitatively a major overestimate because the four-body interaction decays quickly with monomer separation and most four-body interactions are negligible.

In 2010, the precise fitting of 2-body (six atoms) and 3-body (nine atoms) interactions became achievable through the application of permutationally invariant polynomial (PIP) machine-learning regression.^15^ Recently, the PIP approach was extended to handle systems with more than 10 atoms,^16^ and this extension was successfully applied to the 12-atom 4-body water interaction.^17^ This advancement enabled the development of a many-body potential, termed as q-AQUA, which incorporates up to the 4-body interaction using precise PIP fitting of extensive data sets of CCSD(T) energies.^18^ For further details regarding the data sets, fitting details, precision metrics, and numerous tests, we direct interested readers to the aforementioned paper. Before discussing the key performance highlights of the q-AQUA potential for water clusters and liquid water, we remark on the strategy adopted to describe the *n*-body potentials in the long-range regime. In the case of the 2-body interaction, the long-range behavior is represented by the dipole–dipole interaction, utilizing the dipole moment of the isolated water monomer. This approach is justified, since the induction effects that enhance the dipole moment predominantly occur in the short-range regime. The dipole–dipole interaction was obtained based on a high-level dipole moment surface for the flexible water monomer.^19^ Instead of using the known expression for this interaction, we used the widely used Coulomb expression
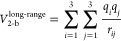
2where *q*_*i*_ and *q*_*j*_ are the partial charges on the *i*th atom of monomer 1 and the *j*th atom of monomer 2, and *r*_*ij*_ is the distance between the atoms. The partial charge on each atom of the water monomer is uniquely obtained from the monomer dipole moment surface. It is perhaps worth emphasizing that these partial charges are not empirically determined, as they are in the widely used rigid-monomer TIP4P and SPC potentials^20,21^ and their extensions for flexible monomers, q-SPC^22^ and q-TIP4P/F.^23^ Of course this interaction must be “damped” to zero in the range where it matches direct CCSD(T) 2-b energies; details of this damping are given in ref (18).

**Figure 1:**
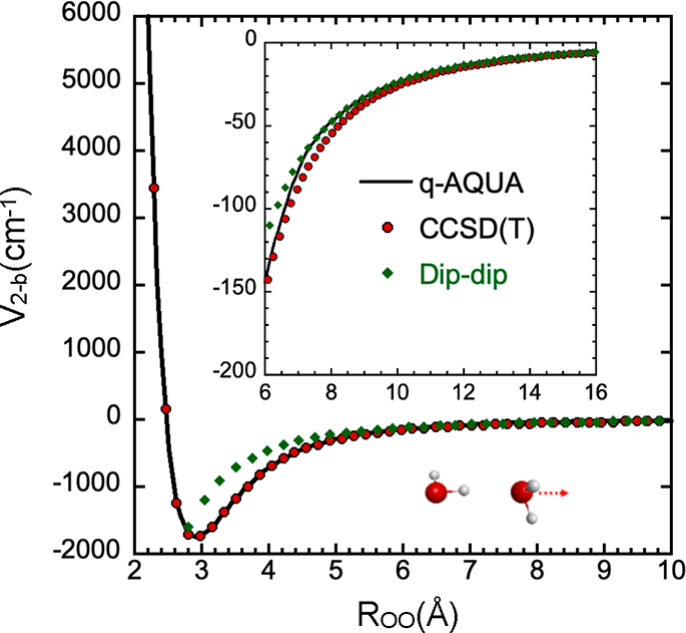
Comparison of the 2-body fit (black line) and direct CCSD(T) energies (red circles) for an attractive cut. The isolated monomer dipole–dipole interaction is also indicated (green diamonds).

**Figure 2:**
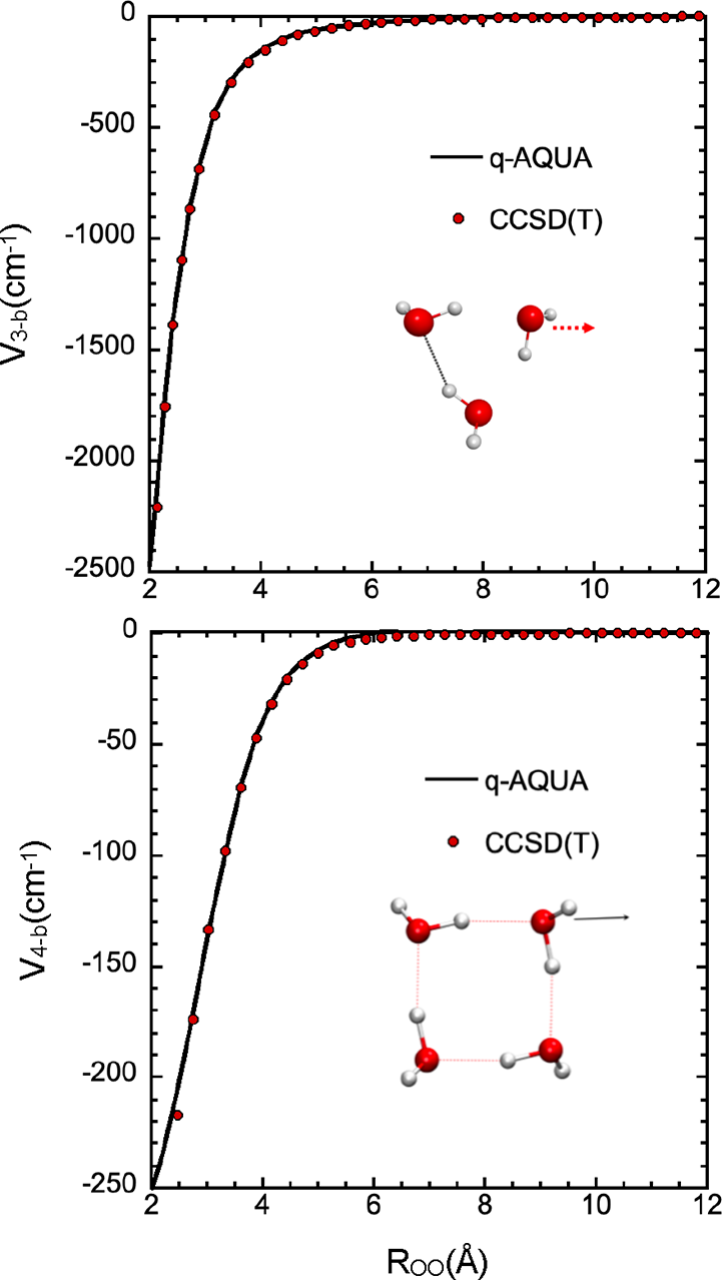
Comparison of 3-b (top) and 4-b (bottom) q-AQUA potentials with direct CCSD(T) energies for an attractive cut.

The long-range 3-body and 4-body interactions are due to induction, which can in principle be obtained using standard, but costly approaches. Since these interactions are both weaker and shorter ranged than the 2-body interaction, our approach was to just rely on fitting the CCSD(T) energies directly and then damp the fit to zero over a finite range. To demonstrate these effects, we show comparisons of the q-AQUA 2-body, 3-body, and 4-body potentials with direct CCSD(T) energies for attractive 1d cuts in Figures 1 and 2, respectively. First, consider the 2-body comparison in Figure 1: the dipole–dipole interaction merges with the CCSD(T) energies at long-range but becomes less accurate at short monomer distances. Interestingly, and as expected, it increasingly underestimates the CCSD(T) energies at a shorter range where polarization effects become increasingly important. Exchange–repulsion interaction effects dominate at distances less than around 3 Å, and the rapid rise in energy occurs at around 2.6 Å. In q-AQUA, the PIP 2-body fit is switched smoothly to the dipole–dipole interaction in the range of 6.5–7.8 Å. Thus, q-AQUA is in excellent agreement with the CCSD(T) energies in both short and long ranges.

Corresponding comparisons for the q-AQUA 3-body and 4-body interactions are shown in Figure 2. Several important points can be highlighted regarding these interactions. The energies associated with the 3-body and 4-body interactions are smaller in magnitude compared to the 2-body interactions. The magnitude of 4-body interactions is generally smaller than the 3-body ones, which also aligns with expectations. Additionally, the range of these interactions is shorter than that of the 2-body interactions, as anticipated. The q-AQUA fits are specifically dampened to zero over different ranges for the 3-body and 4-body interactions, allowing for flexibility and user-defined control over these ranges. These potentials exhibit a rapid shift toward negative values at shorter distances, particularly at 2 Å, where the 2-body interaction is highly repulsive but the 3-body and 4-body interactions are both attractive. The latter does become repulsive at shorter distances.^17^ Lastly, it is worth noting that the 1-body term in q-AQUA corresponds to the spectroscopically accurate potential for H_2_O.^24^

It is important to emphasize that the precise fitting of 2-body and 3-body electronic energies for flexible monomers was done more than a decade ago, beginning with the WHBB potential,^25^ which fit thousands of 2-b CCSD(T) and 3-b MP2 energies using PIPs, followed by MB-pol^26,27^ which fit thousands of 2-b and 3-b CCSD(T) energies using PIPs and CC-pol-flex which fit thousands of 2-b CCSD(T) energies using a multiparameter, physically based functional form for the potential.^28^ MB-pol is the most accurate of this group of three potentials as it uses CCSD(T) data for the 2-b and 3-b energies. It has been used successfully in many applications.^27,29^ As we mention below, MB-pol is a “hybrid” water potential which makes corrections to the TTM4-F potential.^30^ Finally, we note that the size and extent of the CCSD(T) energies used in the new q-AQUA potential are larger than those used in previous potentials. Also, we note again that q-AQUA includes CCSD(T) 4-b energies, unlike those of earlier potentials.

To summarize thus far, q-AQUA is currently the only strictly CCSD(T)-based many (up to 4)-body potential for water that accommodates an arbitrary number of monomers. The feasibility to obtain tens of thousands of *ab initio* CCSD(T) energies for 2–4 body interactions together with precise and efficient PIP fits to the data sets has made this possible. These PIP fits also provide analytical gradients, enhancing the computational efficiency of the approach. q-AQUA has demonstrated its accuracy in describing the energies of hexamer isomers and other water clusters.^18^ Additionally, properties of liquid water, such as radial distribution functions and self-diffusion constants, obtained from NVT classical and path integral simulations employing q-AQUA, have exhibited an accuracy comparable to those obtained using other potentials.

### q-AQUA-pol

Δ-Machine learning is a general term in ML that describes a method to bring an ML representation of a property, determined using low-level theory, to be close to high-level theory, e.g., the CCSD(T) level. For example, the property might be a DFT-based potential energy surface, or it might be a polarizable water force field. Δ-Machine learning has been extensively applied to DFT-based potentials and energies.^31−37^ This subsection describes the very recent realizations of this goal for a water potential, where the underlying low-level potential is the well-known polarizable TTM3-F potential.^38^ Our first effort in this direction^39^ utilized TTM2.1, a semiempirical, many-body force field for water;^40^ however, no applications to condensed phase properties were explored in that study. More recently, we utilized this approach to correct the TTM3-F potential^38^ up to 4-body terms. This latest water potential, derived from the same data sets used to develop q-AQUA, is referred to as q-AQUA-pol.^41^

The expression for the Δ-machine learning q-AQUA-pol potential is a specific example of the general one given previously,^39^ namely
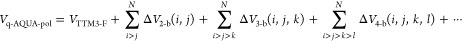
3These correction terms, Δ*V*_*n*-b_, are the differences between the CCSD(T) and TTM3-F *n*-body energies. Ideally, these are short-ranged if TTM3-F^38^ does provide a quantitatively accurate description of the long-range interactions. As has been shown in ref (41), the polarizable TTM3-F force field behaves quantitatively accurately in the long-range against CCSD(T) reference data. Thus, in q-AQUA-pol, we only conducted PIP fits to short-range 2-, 3-, and 4-body interaction corrections. The data set used in q-AQUA-pol is the same as that in q-AQUA where the electronic energies extend to sufficiently high energies. The resulting PES reaches energies well beyond the zero-point energy. This enables the possibilities of quantum simulations to investigate the structural and transport properties of water, ranging from clusters to the condensed phase. Finally, the PES should be invariant with respect to the permutation of monomers, and each monomer should also be invariant with respect to the interchange of two H atoms. This requirement is inherently incorporated through the use of permutationally invariant polynomials in ML fits.

We note that MB-pol^26,29^ also falls under this category of MB approach with a polarizable FF, where the FF is TTM4-F.^30^ The latest version, MB-pol(2023),^42^ also makes use of extensive CCSD(T) energies from the q-AQUA data set.^18^ This updated version is indeed more accurate than previous versions of MB-pol and is certainly at the state-of-the-art.

### Selected Results from q-AQUA and q-AQUA-pol

Here we examine the q-AQUA and q-AQUA-pol potentials for simulations of bulk water properties, as well as the energies of the centrally important isomers of the water hexamer. Specifically, classical molecular dynamics (MD), path integral molecular dynamics (PIMD), and ring polymer molecular dynamics (RPMD)^23,43^ were used to calculate both static and dynamic properties of liquid water. All the MD simulations were performed with the i-PI software.^44^ The computational details about these calculations are provided in the Supporting Information of the original papers.^18,41^

**Figure 3:**
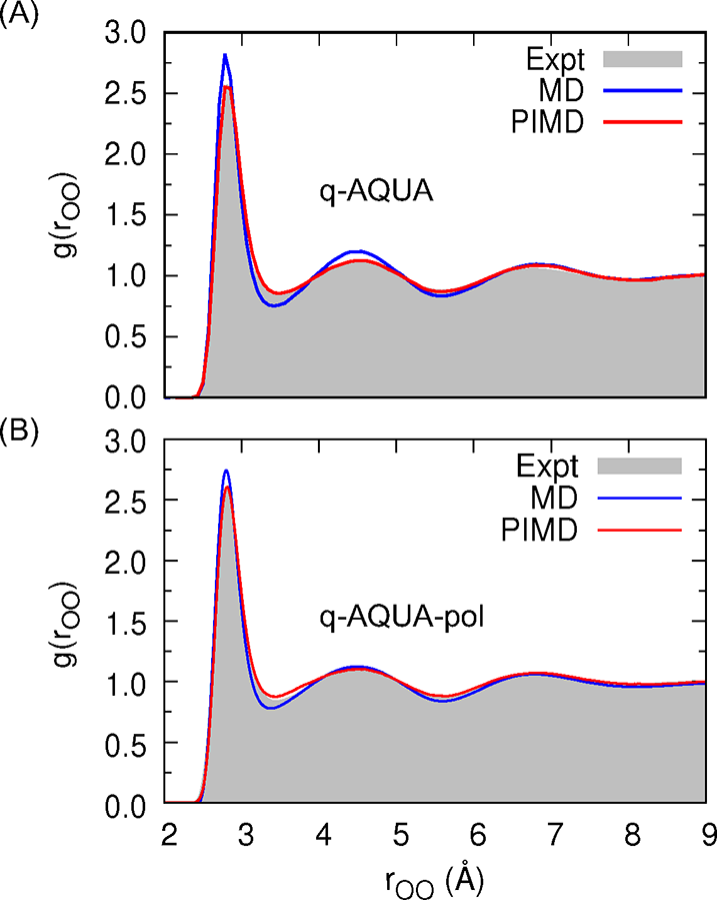
OO radial distribution function from classical (blue) and path integral (red) molecular dynamics simulations at 298 K using q-AQUA (panel A) and q-AQUA-pol potentials (panel B). The experimental data are from refs (45 and 46). Simulation data are from refs (18 and 41).

**Figure 4:**
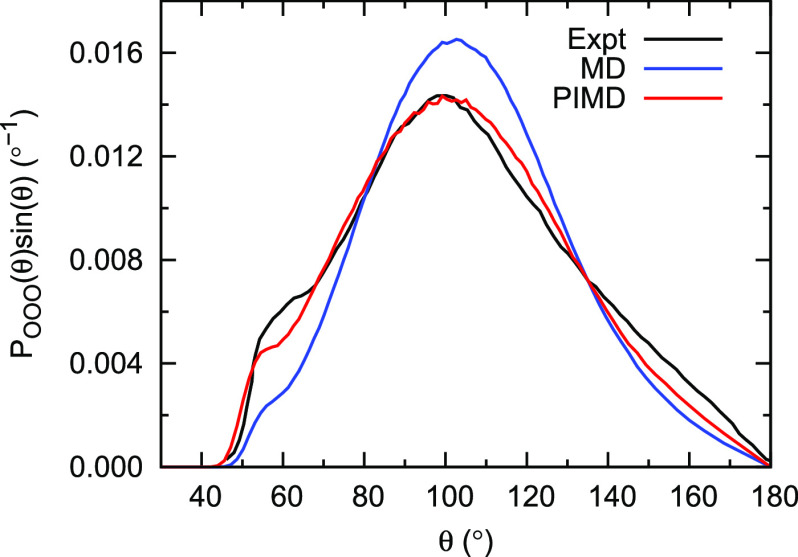
Oxygen–oxygen–oxygen triplet angular distribution functions from classical and path integral molecular dynamics simulations at 298 K and 1 atm using the q-AQUA-pol potential. The experimental data are taken from ref (48). Reproduced from ref (41). Copyright 2023 American Chemical Society.

In panel A of Figure 3, the OO radial distribution functions (RDF) obtained from NVT MD and PIMD simulations at 298 K using the q-AQUA potential are presented. The classical MD simulations yield reasonable predictions for the peak positions of the RDF compared to experimental measurements. However, there is a discrepancy in the amplitudes of the peaks that do not align well with the experimental data. On the other hand, the OO RDF calculated from PIMD simulations exhibits significantly improved agreement with both the peak positions and amplitudes observed in experiments. Similar trends are observed in panel B of Figure 3 from NPT simulations at 298 K and 1 atm, utilizing the q-AQUA-pol potential. The classical MD approach tends to predict more localized water structures, while the inclusion of nuclear quantum effects in PIMD simulations results in a more delocalized OO radial distribution function. The RDFs obtained with just the 2-b terms in q-AQUA and q-AQUA-pol were reported in refs (18 and 41). In those cases, inaccuracies were observed, particularly with the second hydration peak appearing at larger OO distances. Interestingly, as the level of truncation increases from 2-body to 3-body and finally to 4-body interactions, the peaks in the RDFs shift toward shorter OO distances. This suggests the presence of an effective additional attraction when considering higher-order *n*-body interactions. Figure 4 shows the oxygen–oxygen–oxygen (OOO) triplet angular distribution functions of liquid water at 298 K and 1 atm, obtained from simulations with the q-AQUA-pol potential. Consistent with the observations in the OO radial distribution functions, the results from PIMD simulations exhibit considerably better agreement with experimental data compared to the classical MD results. The inclusion of nuclear quantum effects in PIMD simulations improves the accuracy of capturing the triplet angular distribution of water molecules, highlighting the importance of considering quantum effects in accurately describing the behavior of liquid water.

**Figure 5:**
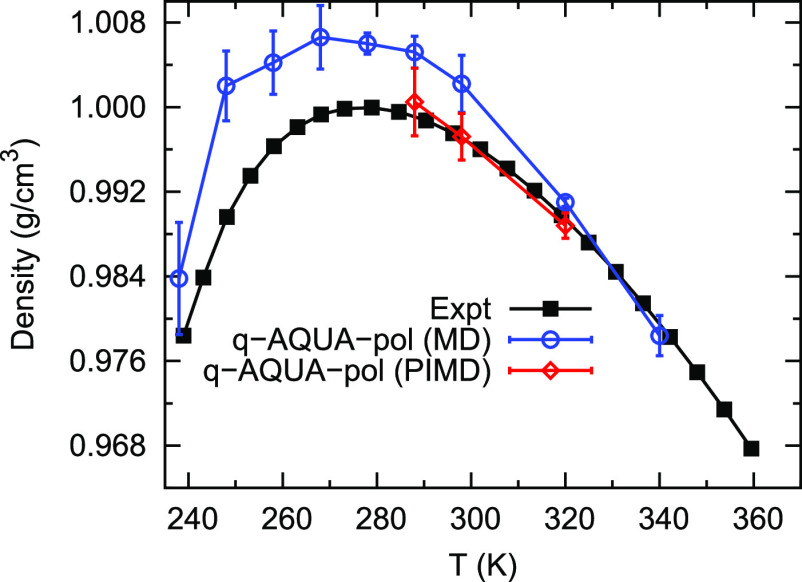
Temperature-dependence of the density of liquid water at 1 atm. The experimental data are taken from refs (50) and (51). Reproduced from ref (41). Copyright 2023 American Chemical Society.

Additional NPT classical MD and PIMD simulations using q-AQUA-pol potential were performed at 1 atm with temperatures from 238 to 340 K. The calculated densities of liquid water are presented in Figure 5. In the high-temperature region, both classical MD and PIMD simulations predict densities that reasonably agree with experimental measurements. As the temperature decreases, the differences between the classical MD predictions and experimental data become more pronounced, although the overall trend still aligns well. This is expected since classical MD neglects the consideration of nuclear quantum effects, which have been demonstrated to be important in water density simulations.^49^ To illustrate the impact of nuclear quantum effects, PIMD NPT simulations were conducted at 288, 298, and 320 K. As observed in Figure 5, the predicted water densities from PIMD simulations exhibit quantitative agreement with experimental data. Furthermore, the improvement over classical MD results becomes increasingly significant as the temperature decreases. Again, this highlights the crucial role of nuclear quantum effects in accurately describing the density of water and emphasizes the advantage of employing PIMD simulations to capture these effects.

**Figure 6:**
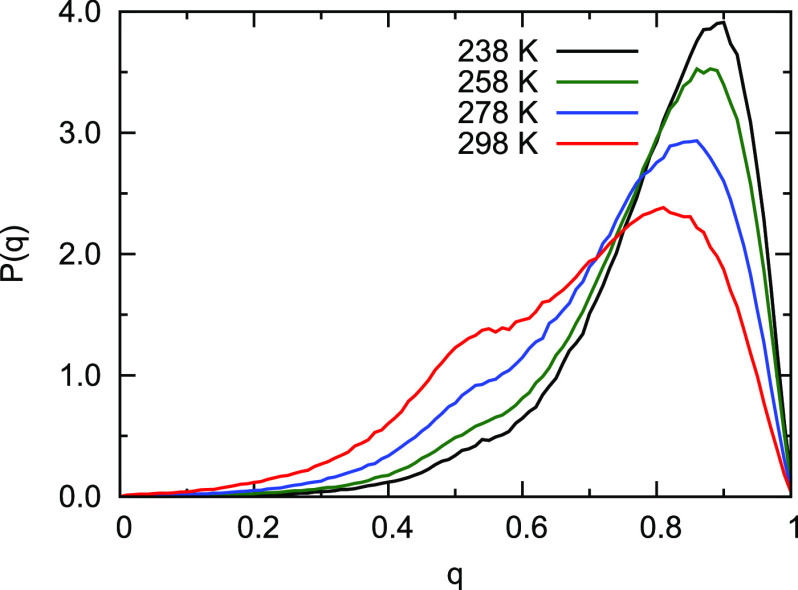
Probability distribution of the tetrahedral order parameter *q* at different temperatures from classical MD simulations. Reproduced from ref (41). Copyright 2023 American Chemical Society.

The tetrahedral order parameter distribution as a function of the temperature is depicted in Figure 6. As seen, as temperature decreases to the supercooled liquid regime, the distribution narrows significantly, indicating more tetrahedral H-bonding structures. In the next section, we show this distribution at 298 K for q-AQUA and one NN-TL potential along with q-AQUA-pol. We also defer presenting results for the self-diffusion constant using q-AQUA and q-AQUA-pol to that section.

**Figure 7:**
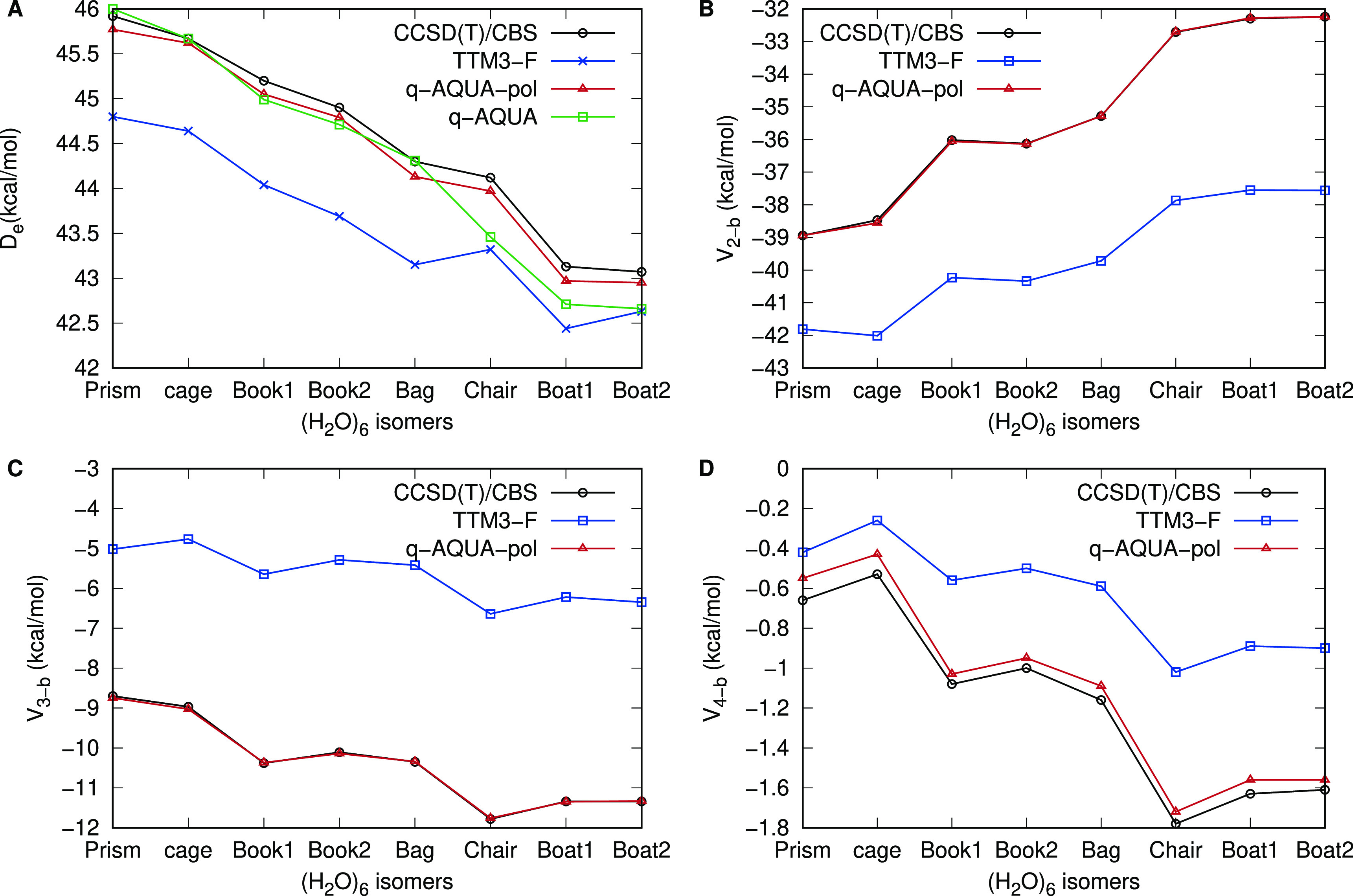
Binding energies (A), 2-body energies (B), 3-body energies (C), and 4-body energies (D) for water hexamer isomers from TTM3-F, q-AQUA (panel A), q-AQUA-pol, and benchmark CCSD(T) calculations (data taken from ref (52)).

Next, we consider the accuracy of q-AQUA and q-AQUA-pol for the centrally important water hexamer binding energies shown in Figure 7. Panel A shows the electronic dissociation energies for eight isomers. It is evident that q-AQUA-pol achieves nearly perfect agreement with the CCSD(T)/CBS results, which is an unprecedented level of accuracy for a water potential. q-AQUA is also highly accurate, except for the three highest energy isomers. Both potentials exhibit significantly higher accuracy compared to TTM3-F.^38^ However, it should be noted that q-AQUA-pol, being a Δ-ML correction to TTM3-F, benefits from the many-body polarization effects beyond the 4-body level present in TTM3-F, which accounts for its improved accuracy for the chair and two boat isomers. Panels B, C, and D provide detailed comparisons of the many-body contributions to the electronic dissociation energies up to the 4-b level. It can be observed that the 4-b energies have the largest magnitude for the chair and boat isomers.

In summary thus far, both q-AQUA and q-AQUA-pol demonstrate high accuracy in predicting condensed phase properties of water as well as the binding energies of water hexamer isomers. However, q-AQUA-pol incorporates polarization effects beyond the 4-b level through TTM3-F, thereby enhancing its accuracy.

**Figure 8:**
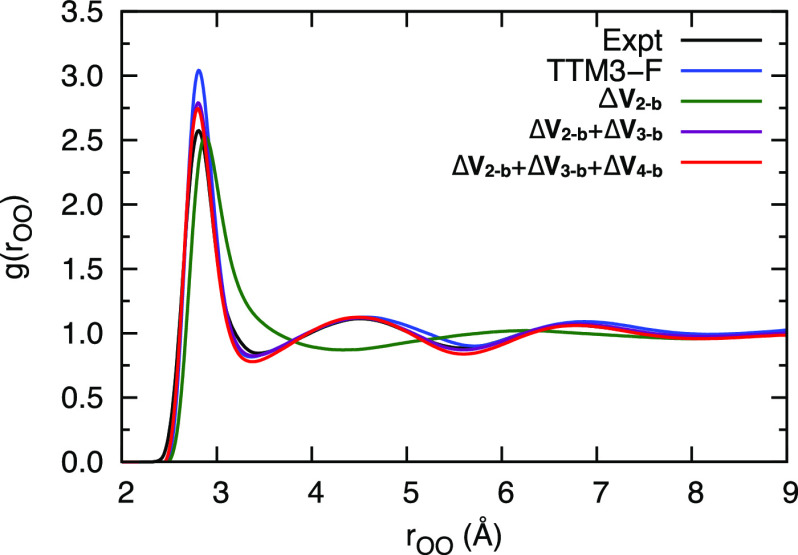
Effects of 2-b, 3-b, and 4-b corrections on the OO radial distribution function of liquid water at 298 K from classical MD simulations. The experimental data are taken from refs (45 and 46).

Before discussing other approaches to bring a low-level water potential to the CCSD(T) level, it is of interest to examine the corrections to TTM3-F term by term. This is shown for the OO radial distribution function at 298 K using NVT calculations (Figure 8). As seen, the RDF using TTM3-F is in good agreement with experiment. However, adding the CCSD(T) 2-b correction Δ*V*_2-b_ results in an RDF that is significantly less accurate than the TTM3-F one. By adding the 3-b correction agreement with experiment is very good. The 4-b correction makes a small contribution to this property. These results are shown in order to make the following point. Namely, we advise caution when correcting a force field using just a 2-b *ab initio* correction, even at the CCSD(T) level. Doing so may result in a decrease in accuracy. Based on extensive work on the FF for water, for which *ab initio* 3-b interactions are essential, we recommend investigating these interactions for correcting FFs.

## Transfer and Δ-Learning

Δ-Learning has also been applied by another group to develop a coupled-cluster level PES of water,^53^ although it differs from q-AQUA-pol and MB-pol(2023) in that the Δ is not learned in the many-body manner. In addition to Δ-learning, transfer learning (TL) is a method to modify the optimized parameters of a machine-learned potential trained on, say, low-level DFT energies/forces using a relatively small training set of CCSD(T) energies.^54^ This approach was recently applied for a neural network (NN) potential for water.^55^ Both PESs use the basic Tersoff form of the potential, i.e., as a sum of atomic energies each of which is element-specific and given by a general density expression that contains radial and angular components.^56^ Behler and Parrinello made a major significant modification to this basic form by training a NN for the atomic energy using DFT energies.^57^

In the recent TL PES, this form of the potential was trained on DFT or Hartree–Fock energies and forces of 16 water monomers in a periodic box based on configurations selected using an active learning procedure.^55^ Then the transfer-learning approach was applied using a small data set of (periodic) CCSD(T) energies for 16 monomers. The NN-Δ-ML water potential^53^ was first trained on domain-based local pair natural orbital (DLPNO) MP2 energies and forces, using finite clusters of 64 water monomers sampled from AIMD. Then the difference between the DLPNO-CCSD(T) and DLPNO-MP2 interaction is fit by a second NN as the correction to the MP2-level NN. This PES additionally makes use of fixed-charged electrostatic interactions that are damped as usual in the short-range and repulsive Yukawa potentials. The charges are −0.8 for O and 0.4 for H, exactly the ones in the first generation (1981) fixed charged model, TIP.^58^ The interested reader is referred to refs (53) and (55) for more details.

The differences between these two approaches and the many-body expansion (MBE) approach used in q-AQUA, q-AQUA-pol, MB-pol, and MB-pol(2023) are noteworthy. These approaches directly fit the energies and forces of relatively large water clusters (16 monomers in a periodic box and 64 monomers), sampled from large-scale AIMD or on-the-fly PIMD simulations. In contrast, the MBE approach involves fitting electronic energies of small clusters, specifically the dimer of the 2-body interaction, the trimer for the 3-body interaction, and the tetramer for the 4-body interaction. These small cluster data sets are extensive, and the associated energies are typically computed using the CCSD(T) method. The data sets for small water clusters cover a broader range of energies than those obtained from AIMD and PIMD simulations at around 300 K, which are used in those fits. While this difference may not be crucial for MD and PIMD simulations conducted at room temperature, it can become significant for quantum simulations that explore high-energy regions of the PES. Interested readers can refer to a perspective on the differences in data sets obtained from MD simulations at 300 K and higher energies for more details on this topic.^59^ Overall, the Δ-ML and TL approaches directly incorporate information from large-scale simulations, while the MBE approach focuses on fitting small cluster energies at the CCSD(T) level. Both approaches have their advantages and considerations, and the choice is dependent on the specific requirements in the fitting architecture.

**Figure 9:**
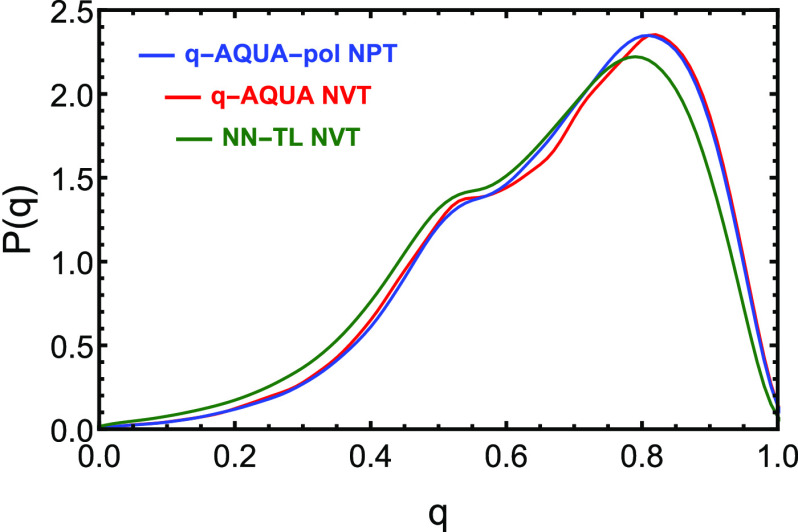
Probability distribution of the tetrahedral order parameter *q* at room temperature from q-AQUA-pol, q-AQUA, and recent NN-transfer-learned potential with CCSD(T) accuracy.^55^ Reproduced from ref (41). Copyright 2023 American Chemical Society.

The above remarks notwithstanding, it does appear that the MBE and Δ-ML/TL potentials produce results of similar accuracy for several condensed phase properties. For example, Figure 9 demonstrates that both the MBE potentials (q-AQUA and q-AQUA-pol) and the TL potential (NN-TL) yield similar descriptions of the local structure of water molecules, characterized by the tetrahedral order parameter. Such good agreement among all three potentials indicates convergence between the MBE and TL approaches in predicting liquid-phase water structures. As shown in Table 1, the self-diffusion coefficients, a measure of the mobility of water molecules, also exhibit good agreement with experimental values for these different types of potentials. It is important to note that the nuclear quantum effects play a significant role in the dynamical properties of water, including self-diffusion. In the case of q-AQUA and q-AQUA-pol potentials, the inclusion of NQE through the TRPMD method leads to significantly larger self-diffusion coefficients compared to classical MD simulations. This is consistent with the understanding that NQE weakens the hydrogen bonding network and promotes molecular motion in liquid water. Interestingly, the NN-TL potential exhibits a less pronounced impact of NQE on self-diffusion coefficients. This may arise from the competing effects of intramolecular and intermolecular NQE in liquid water, as discussed in ref (23). The intramolecular NQE weakens covalent bonds and enhances molecular dipole moments, while intermolecular NQE weakens hydrogen bonds and forms a less structured hydrogen-bonded network. In the case of q-AQUA and q-AQUA-pol, the dominance of intermolecular NQE leads to larger diffusion coefficients. However, in the NN-TL potential, there appears to be a balance between these two types of NQE, resulting in only a slight increase in diffusion coefficients.

**Table 1:** Self-Diffusion Coefficient, *D* (Å^2^/ps), of Liquid Water at 298 K from Classical and Quantum Simulations with Different Potentials

Potential	Classical	Path integral	Expt.^a^
q-AQUA	0.145 ± 0.012	0.226 ± 0.020	0.230
q-AQUA-pol	0.185 ± 0.004	0.233 ± 0.027	
NN-Δ-ML^b^		0.244 ± 0.002	
NN-TL^c^	0.221 ± 0.006	0.230 ± 0.008	

a From refs (60) and (61).

b From ref (53).

c From ref (55), and simulations were conducted at 300 K.

Thus far, we have shown that the efforts at transfer learning were successful in predicting condensed-phase properties of water including radial distribution function, self-diffusion coefficient, and tetrahedral order distribution functions. The specific choice between the MBE and TL approaches may depend on factors such as computational efficiency, desired level of accuracy, and the ability to capture different aspects of water’s behavior. The computational scaling advantages of NN-TL potentials, achieved through the use of the sum of atomic energies, make them appealing for large molecular and materials systems. However, there are certain aspects of these new potentials that have yet to be fully explored, such as their performance on water clusters. Experimental studies have extensively investigated water clusters, providing important tests for theoretical models and yielding valuable insights into the nature of hydrogen bonding in water.^62−66^ It is reasonable to speculate that the accuracy of the NN-TL potentials may not be on par with that of q-AQUA and q-AQUA-pol when it comes to describing water clusters. This is primarily due to the lack of direct training on clusters in the DFT and TL fitting process. The absence of explicit consideration for the unique properties and behavior of water clusters during PES development could potentially limit the accuracy of NN-TL potentials in this regime. To gain a more comprehensive understanding of the performance of NN-TL potentials on water clusters, it would be beneficial to assess their capabilities in reproducing the structural, energetic, and spectroscopic properties observed in the experimental studies.

## Timing

Finally, it is worth considering the computational cost associated with using q-AQUA and q-AQUA-pol potentials for dynamics simulations of a 256-water system. All tests were conducted using single or multiple cores of a 2.4 GHz Intel Xeon processor. The timing results for the q-AQUA and q-AQUA-pol potentials are summarized in Table 2.

**Table 2:** Computational Costs of the q-AQUA and q-AQUA-pol Potentials for Energy and Gradient Calculations of a 256-Water System

q-AQUA					
		Time for energy (s)		Time for energy+gradient (s)	
Component	Number	1 core	8 cores	1 core	8 cores
*V*_1-b_	256	0.002	0.002^a^	0.003	0.003^a^
*V*_2-b_	32640	0.23	0.02	0.72	0.08
*V*_3-b_	84051	0.42	0.05	1.94	0.26
*V*_4-b_	115922	1.26	0.17	4.34	0.52
Total		2.00	0.35	7.12	0.97

q-AQUA-pol					
		Time for energy (s)		Time for energy+gradient (s)	
Component	Number	1 core	8 cores	1 core	8 cores
TTM3-F	/	NA	NA^b^	0.27	0.07^b^
Δ*V*_2-b_	3816	0.11	0.02	0.36	0.06
Δ*V*_3-b_	20790	0.65	0.23	2.20	0.39
Δ*V*_4-b_	28786	0.30	0.04	1.05	0.14
Total		1.33	0.36	3.88	0.66

a 1-b terms are not parallelized.

b The current TTM3-F force field code calculates the energy and gradient simultaneously by default.

As shown in Table 2, the computation of 4-body interactions is the most computationally intensive part of the q-AQUA potential, accounting for more than half of the computation time in both energy and gradient calculations. It is important to note that the number of final calculated four-body terms, 115922, is only a small fraction of the total four-body interactions, which would be on the order of 256^4^ in a 256-water system. This is due to the fast damping of 4-body interaction to 0 when the monomer distances exceed 7.5 Å in the q-AQUA potential. By directly dampening high-order interactions beyond this distance, significant computational savings are achieved. Additionally, efficient OpenMP parallelization allows for a speed-up of up to 6 times when using 8 cores. Furthermore, the cost of gradient calculations is observed to be approximately twice the cost of energy calculations, benefiting from the efficient implementation of reverse differentiation in the PIP approach.^16^

Similar trends in the computational cost are observed for the q-AQUA-pol potential. The overall cost of energy or energy+gradient calculations with q-AQUA-pol is approximately half that of q-AQUA. This is primarily because the total number of correction terms (Δ*V*_2-b_, Δ*V*_3-b_, Δ*V*_4-b_) is much smaller than the corresponding *n*-body interactions in q-AQUA. The implementation of finite-range switching functions enables the selection of a short O–O region for corrections, resulting in a reduced number of these interaction terms. For example, only 20790 Δ*V*_3-b_ terms need to be calculated with a maximum O–O distance smaller than 7.0 Å. The accuracy of the TTM3-F force field in describing long-range many-body interactions allows for a safe selection of this short O–O region for corrections.

It is evident from Table 2 that the calculation of Δ*V*_3-b_ interaction terms accounts for the majority of the total computation time in q-AQUA-pol. A smaller cutoff range leads to a significant reduction in the number of 3-b correction terms. However, this reduction comes at the expense of some accuracy. We investigate the impact of the cutoff maximum O–O distance of 3-b interactions on the cost and accuracy of the q-AQUA-pol potential. The calculated computational cost associated with energy and gradient calculations in q-AQUA-pol for the same 256-water system over a range of maximum O–O distances are given in Table 3. Note that the cost for the Δ*V*_2-b_ correction terms and TTM3-F force field remains the same as in Table 2 throughout these calculations. It can be seen from Table 3 that if we only take 3-b interactions with a maximum O–O distance smaller than 6.0 Å into account, the total number of Δ*V*_3-b_ correction terms is reduced to 8266, and the cost of 3-b interaction calculation for the energy alone and for the energy and gradient speed up by a factor of 2.2.

**Table 3:** Computational Cost of the 3-b Interaction of q-AQUA-pol Potential for Energy and Gradient Calculations of a 256-Water System

Maximum *r*_OO_ (Å)	Δ*V*_3-b_ term	Time for energy (s) (1 core)	Time for energy+gradient (s) (1 core)
6.0	8266	0.30	0.99
6.3	10972	0.37	1.25
6.5	13369	0.43	1.49
6.7	15825	0.50	1.73
7.0	20790	0.65	2.20

**Figure 10:**
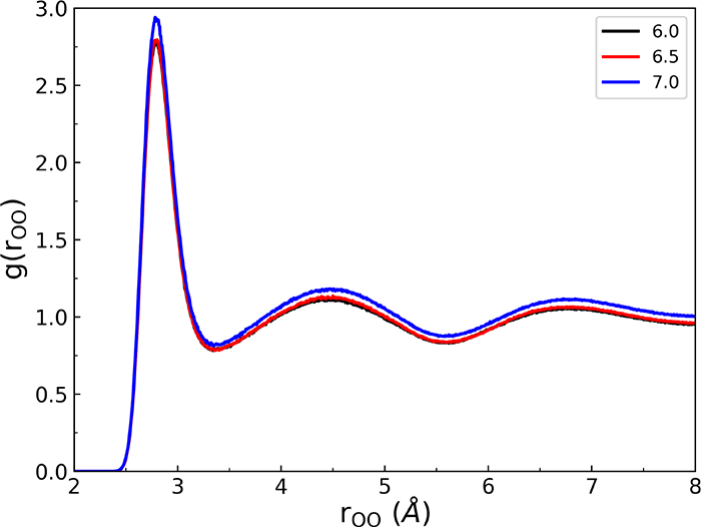
OO radial distribution function from classical MD simulations at 298 K using q-AQUA-pol potential for three range parameters indicated (Å) for the 3-b interaction.

**Figure 11:**
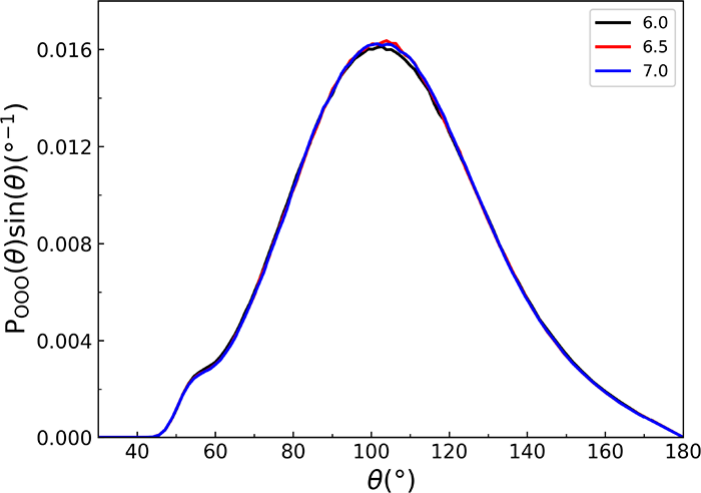
Oxygen–oxygen–oxygen triplet angular distribution functions of liquid water at 298 K from classical MD simulations for three range parameters indicated (in Å) for the 3-b interaction.

**Figure 12:**
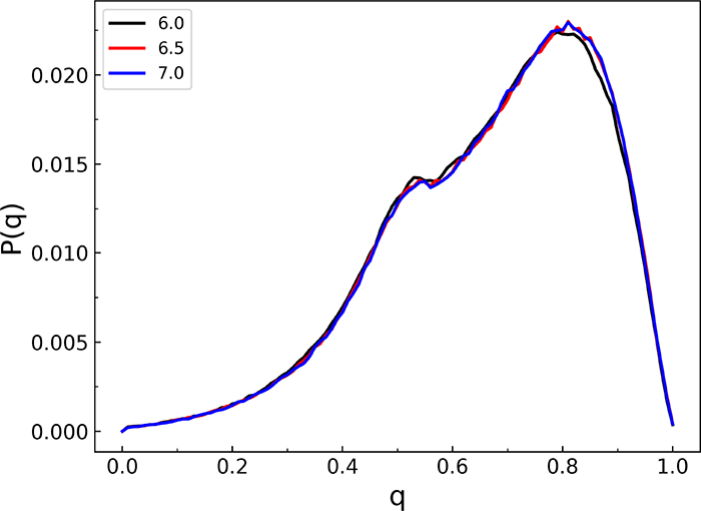
Probability distribution of the tetrahedral order parameter *q* at 298 K from classical MD simulations for three range parameters indicated (in Å) for the 3-b interaction.

To investigate the influence of the cutoff distance on condensed phase properties, we conducted classical MD simulations of liquid water at a temperature of 298 K and pressure of 1 atm using the q-AQUA-pol potential. Figure 10 displays the radial distribution functions (RDFs) for oxygen–oxygen (O–O) pairs obtained from the MD simulations with three different cutoff distances for the three-body (3-b) interactions. Although the overall RDF for a cutoff distance of 7 Å exhibits a slightly higher magnitude compared to the other two cutoff values, the positions of the first and second peaks, which correspond to H-bonded neighbors and non-H-bonded water molecules, respectively, remain consistent across all three cutoff distances. This indicates that the local structure and H-bonding network of liquid water are preserved regardless of the range chosen for the 3-b interactions. Furthermore, we analyzed the oxygen–oxygen–oxygen (O–O–O) triplet angular distribution function P_OOO_(θ) and tetrahedral order parameter at room temperature for three different cutoff distances in order to determine the influence of the cutoff distance. The corresponding results are shown in Figures 11 and 12, respectively. It is evident from these figures that the computed angular distribution function and tetrahedral order parameter are nearly independent of the chosen cutoff distance. Again, this suggests that the orientational ordering and local structure of liquid water are not significantly affected by the specific range used for the 3-body interactions.

By utilizing multiple cores and adjusting the ranges of the 3-b and 4-b interactions, the computational timings can be greatly modified. It is important to note that the choice of cutoff distances should be carefully considered, depending on the property of interest. The convergence of results can be verified by varying the ranges until the desired properties are satisfactorily converged. Overall, these findings demonstrate the flexibility of the q-AQUA-pol potential in terms of adjusting the computational parameters to achieve accurate and efficient simulations of liquid water.

## Summary and What’s Next

The status of the CCSD(T)-based general potentials for water is that they have “arrived”. These basically come in three versions. One, q-AQUA, is based on a strict many-body representation truncated at the 4-b term. The second is where we employ a Δ-ML approach, where a sophisticated polarizable potential is corrected using the MB approach. This was demonstrated by q-AQUA-pol which corrected TTM3-F up to the 4-b terms. MB-pol(2023) falls into this category but uses TTM4-F. These “bottom up” potentials produce highly accurate results from clusters to the condensed phase. The third version is based on ML fitting of DFT energies of 16 and 64 monomers obtained from molecular and path integral molecular dynamics in NVT simulations at 300 K. These initial fits are then corrected using Δ-learning and transfer learning with a limited number of CCSD(T) energies. Results for the condensed phase properties are accurate and comparable to those obtained with q-AQUA, q-AQUA-pol, and MB-pol(2023). However, no specific tests on water clusters using these Δ-learning and transfer learning potentials have been reported at this stage.

It is worth mentioning that another CCSD(T)-based potential for water is being developed by Zhang and collaborators, utilizing the fundamental invariant neural network (FINN) approach^67,68^ along with extensive high-quality data sets. The preliminary version of this many-body potential includes fits on 220,000 CCSD(T)/CBS 2-body data and 430,000 CCSD(T)/aVTZ 3-body data. This potential has shown promising results and has been successfully applied to torsional tunneling splitting calculations of water trimers.^69^

Looking ahead, these potentials can be used in many possible applications and hopefully with some confidence in their predictive accuracy. Examples from our group include diffusion Monte Carlo calculations, which were already reported for q-AQUA and q-AQUA-pol to small clusters,^18,41^ as well as semiclassical initial value representation dynamics calculations for spectroscopy and vibrational wave functions.^70,71^ To be run successfully, these methods need a potential energy surface that is reliable at energies even higher than the zero-point one. Of course, this requires that these potentials be made available. Source code for q-AQUA and q-AQUA-pol are available on github.^47,72^
